# Effects of vitamin D3 supplementation on strength of lower and upper extremities in athletes: an updated systematic review and meta-analysis of randomized controlled trials

**DOI:** 10.3389/fnut.2024.1381301

**Published:** 2024-05-27

**Authors:** Qi Han, Mai Xiang, Nan An, Qiushi Tan, Jing Shao, Qirong Wang

**Affiliations:** ^1^Sports Nutrition Center, National Institute of Sports Medicine, Beijing, China; ^2^Key Lab of Sports Nutrition, State General Administration of Sport of China, Beijing, China; ^3^Sport Science College, Beijing Sport University, Beijing, China; ^4^National Testing & Research Center for Sports Nutrition, Ministry of Science and Technology of the People’s Republic of China, Beijing, China

**Keywords:** exercise, muscle, 25(OH)D, strength, meta-analysis

## Abstract

**Background:**

Coaches and athletes are increasingly interested in understanding athletes’ serum vitamin D levels, their impact on strength, physical performance, and athletic outcomes. Previous meta-analyses were reported with limited sample size and no significant overall effect was found. Hence, it is crucial to conduct a thorough and up-to-date systematic examination and meta-analysis to elucidate the potential advantages of supplementing with vitamin D3 in enhancing muscle strength for athletes.

**Methods:**

We performed a thorough investigation, spanning three databases (PubMed, EBSCO, and Cochrane Library), seeking randomized controlled trials (RCTs) in all languages. These trials delved into the influence of vitamin D3 supplementation on the changes of pre- and post-intervention muscle strength in healthy athletes. Our systematic examination and meta-analysis took into account serum 25(OH)D levels exceeding 30 ng/mL as a marker of adequacy.

**Results:**

Ten RCTs, comprising 354 athletes (185 in the vitamin D3 group and 169 in the placebo group), fulfilled the inclusion criteria. During the study, 36 athletes were lost to follow-up, leaving 318 athletes (166 in the vitamin D3 group and 152 in the placebo group) with documented complete results. In comparison with the placebo group, there is a significant increase between the changes of pre- and post-intervention serum 25(OH)D levels among athletes following a period of vitamin D3 supplementation (MD 14.76, 95% CI: 8.74 to 20.77, *p* < 0.0001). Overall effect of four strength measurements including handgrip, one repetition maximum Bench Press (1-RM BP), vertical jump, and quadriceps contraction was not significantly improved (SMD 0.18, 95% CI: −0.02 to 0.37, *p* = 0.08), but there was a significant increase in quadriceps contraction (SMD 0.57, 95% CI: 0.04 to 1.11, *p* = 0.04).

**Conclusion:**

This updated meta-analysis indicates the potential benefits of vitamin D supplementation for enhancing muscle strength in athletes when analyzing its quantitatively synthesized effects. With limited available studies for the quantitative synthesis, it cannot warrant significant overall enhancements in muscle strength when athletes attain adequate serum 25(OH)D levels through supplementation.

## 1 Introduction

Vitamin D constitutes a vital group of substances essential for upholding optimal body functions of living creatures (1). Its primary role lies in facilitating calcium absorption, a fundamental element for bone health (2). Over the past century, extensive research has illuminated the detrimental consequences of vitamin D deficiency, including conditions like osteoporosis (1–3), muscle discomfort, and weakness (4). Lately, there has been a noticeable surge of interest in the examination of vitamin D within the realm of sports science, owing to its purported beneficial impacts on physical fitness, the integrity of skeletal structure, and muscular function (5, 6).

Whether obtained from diet, exposure to sunlight, or through supplementation, both vitamin D2 and D3 can undergo conversion into 25-hydroxyvitamin D [25(OH)D] within the liver, and subsequently, its concentration is assessed in the bloodstream (7). This 25(OH)D can further undergo a conversion process, resulting in the formation of the biologically active compound called calcitriol [1,25(OH)2D], which occurs in the kidneys (8). 1,25-dihydroxyvitamin D3 [1,25(OH)2D3] assumes a critical role in augmenting the absorption of calcium and phosphate in the intestines and stimulating the generation of neogenesis bone tissue (8, 9). Research involving animals has shown that mice deficient in the vitamin D receptor (VDR) exhibit compromised bone health and diminished muscle function, underscoring the significance of VDR in living organisms (9).

In this investigation, we adhered to the guideline which deems a serum concentration of 25(OH)D above 30 ng/mL as satisfactory, aligning with established recommendations (10, 11). It is widely believed that maintaining adequate serum 25(OH)D levels can have a positive impact on athletic performance (12). Nevertheless, attention has been raised regarding the possibility of toxicity when the serum concentration of 25(OH)D surpasses 100 ng/mL (13). The precise mechanism by which it enhances athletic performance continues to be a subject of ongoing research. A suggested mechanism revolves around the increased presence of 1,25(OH)2D and the presence of vitamin D receptors (VDR) within muscle cells, which could conceivably impact the efficiency of calcium binding during muscle fiber contractions (12). In the long run, 1,25-dihydroxyvitamin D may potentially contribute to the enlargement and multiplication of fast-twitch muscle fibers (14, 15) and accelerate the process of lipolysis within the tricarboxylic acid (TCA) cycle (16).

Reports have suggested that athletes frequently experience a notable prevalence of vitamin D insufficiency. This can be attributed to factors such as their heightened metabolic rate, year-round indoor training, limited exposure to ultraviolet rays from sunlight, and a lack of proper measures to monitor and uphold adequate serum 25(OH)D levels because of their strenuous physical activities (17–20). Coaches, athletes, athletic trainers, and healthcare professionals in the realm of sports are growingly preoccupied with the sufficiency of vitamin D in athletes and its potential connection to strength, conditioning, and their overall athletic performance. Previous systematic examinations of vitamin D status and its influence on muscle strength have usually encompassed small-scale trials characterized by relatively modest effect sizes (21–23). Furthermore, most of the existing reviews summarized the impact of vitamin D on muscle function and its relevance to the performance of athletes rather than based on quantitatively synthesis method (24, 25). Based on our previous systematic review and meta-analysis of randomized controlled trials (RCTs) published up until May 2019 (26), it’s crucial to highlight that once serum concentrations of 25(OH)D reach adequate levels through supplementation, there are no noteworthy enhancements in muscle strength with limited sample size at that time. Nonetheless, the correlation between serum vitamin D levels and strength, physical performance, and athletic results remains a subject of significant interest for experts in this field. To offer the most recent and thorough evaluation of the potential influence of vitamin D3 supplementation on muscle strength in athletes, we have carried out a contemporary review and meta-analysis. This builds upon our previous systematic review from 2019 (26) and introduces new parameters for evaluating muscle strength, including vertical jump and grip strength.

## 2 Materials and methodology

### 2.1 Research design

To ensure a robust methodology and comprehensive reporting, this systematic review and meta-analysis rigorously followed the guidelines set forth in the PRISMA (Preferred Reporting Items for Systematic Reviews and Meta-Analysis) statement (27).

### 2.2 Eligibility criteria

Our study employed the PICO approach as follows: Population (P) encompassed healthy male and female athletes aged 10–45 years old engaged in various sports. Intervention (I) involved administering vitamin D3 orally, without specifying dosage or duration. Comparison (C) entailed comparing the intervention group with a placebo group. The outcomes (O) of interest were the Vitamin D3 supplementation impacts on serum 25(OH)D levels and muscle strength.

Our inclusion criteria were limited to RCTs involving athletes, while exclusion criteria encompassed non-randomized trials, studies lacking complete text, trials involving non-athletes, the use of vitamin D2, trials without assessments of muscle strength, and trials involving multivitamin supplementation. Additionally, we excluded studies that involved Paralympic athletes and athletes with medical conditions that could potentially affect their serum 25(OH)D levels or influence their responses to vitamin D3 supplementation. Furthermore, studies were excluded if they incorporated interventions that could influence serum 25(OH)D levels apart from vitamin D3, if they lacked adequate quality-related information, or if they presented incomplete outcome data.

### 2.3 Approaches employed to locate relevant research papers

We performed a comprehensive literature search across the PubMed, EBSCO SPORTDiscus, and Cochrane Library databases, encompassing articles from their inception through September 27, 2023. The search utilized a combination of terms and medical subject headings (MeSH), which included various forms of vitamin D, supplementation, muscle-related keywords, and terms associated with athletic performance. To ensure the quality of the results, duplicates were eliminated during the initial title and abstract screening phase.

### 2.4 Assessment of eligibility, selection of studies, and evaluation of study quality

The screening, selection, and quality assessment of trials were carried out using the PRISMA flow diagram and the Cochrane risk of bias (ROB) assessment tool. The studies were initially screened following the PRISMA checklist, involving an independent review of titles and abstracts by two authors to determine eligibility. Subsequently, two independent authors meticulously assessed the full texts of these articles, taking into account their methodological quality, outcomes, and the possibility of duplication. Any discrepancies that emerged were resolved through mutual agreement or consensus between the authors.

### 2.5 Data retrieval

Two authors independently carried out the extraction of data, with any discrepancies being resolved through consensus. The extracted information encompassed several aspects, including the athletes’ baseline characteristics such as age, gender, living latitude, and sports activities. Additionally, details regarding the vitamin D3 supplementation were collected, including units, dosages, product used, and duration. For consistency, the varied dosages of vitamin D3 supplementation across trials were converted to a daily dosage in international units (IU), with the conversion of 100 IU being equivalent to 2.5 μg. Serum 25(OH)D levels, originating from different trials, were consistently reported in ng/mL, where 1 ng/mL is equivalent to 2.5 nmol/L. Standard deviation (SD) data were obtained from various sources, including ranges, standard errors, confidence intervals (CIs), or *p*-values, in cases where they were not explicitly reported.

### 2.6 Data segmentation and subdivision

During the data extraction process, it came to our attention that nine trials were carried out during the winter, a period characterized by minimal sunlight exposure, while only one trial took place during the fall. The durations of interventions in various studies ranged approximately from 1 to 12 weeks. To ensure consistency, we stratified the trials based on the athletes’ baseline vitamin D status to observe the effects of vitamin D3 supplementation on serum 25(OH)D levels. Moreover, due to the utilization of various methods for measuring muscle strength in the included RCTs, we created subgroups for muscle strength outcomes, categorizing them according to the specific muscle strength tests employed.

### 2.7 Data synthesis

Our analysis involved several steps. Initially, we examined the baseline mean differences (MD) between the vitamin D3 group and the placebo group before supplementation. For comparing the baseline 25(OH)D levels between these two groups, we utilized a random-effects model and applied the inverse variance method to compute the MD in serum 25(OH)D levels. During the post-supplementation intervention phase, we utilized a random-effects model to compute the results. When assessing the results of muscle strength tests, we applied a random-effects model and utilized the inverse variance method to calculate the standardized mean differences (SMDs) between the vitamin D3 supplementation group and the placebo group for various indicators. To assess heterogeneity among studies, we employed Cochran’s Q test, with a significance level of presenting heterogeneity set at *p* < 0.10, where *I*^2^ values less than 40% were considered as low heterogeneity, values between 40 and 60% as moderate, and values exceeding 60% as indicating substantial heterogeneity. We conducted these analyses using Review Manager 5.4 software (28), and generated funnel plot to access the presence of public bias. The statistical significance of overall effect was set at *p* < 0.05.

## 3 Results

### 3.1 Assessment of eligibility and selection of articles

**Figure 1:**
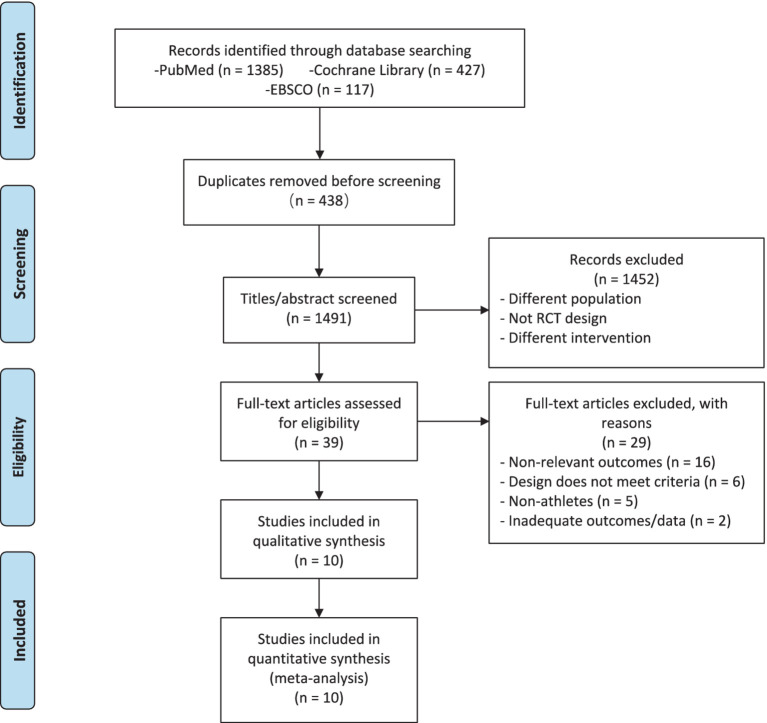
PRISMA flow diagram of the exploration and process of choosing materials.

Figure 1 provides an overview of the search and selection process. Initially, a total of 1,929 titles and abstracts were reviewed. Subsequently, 39 articles were chosen for a comprehensive full-text article review. Out of these 39 articles, 10 RCTs satisfied the inclusion criteria and were consequently incorporated into this meta-analysis.

#### 3.1.1 Publication bias

**Figure 2:**
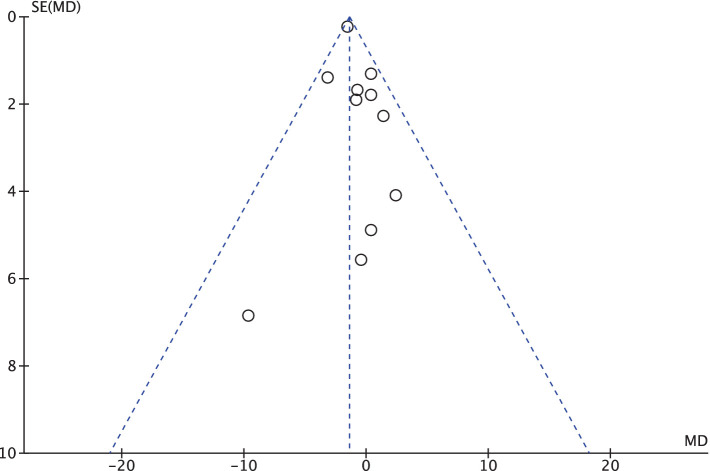
Presents a funnel plot that visualizes the publication bias of baseline serum 25(OH)D levels between different studies.

Figure 2 displays the funnel plots, visually depicting baseline serum 25(OH)D levels between the intervention and placebo groups at the outset of each trial (29–38). On the horizontal axis, the MD values stands for mean difference of serum 25(OH)D. The outcomes of the comprehensive heterogeneity assessment for all the chosen trials reveal minimal heterogeneity (*I*^2^ = 0%, *p* = 0.55). This implies that the likelihood of selection and publication bias exerting an impact on the results of this systematic review and meta-analysis is minimal.

#### 3.1.2 Assessment of bias risk

**Figure 3:**
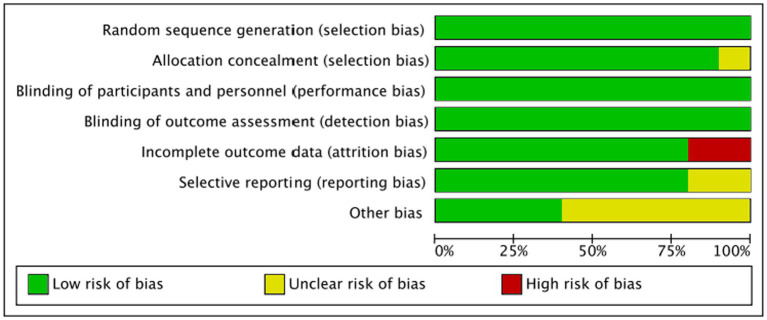
The Cochrane risk of bias assessment.

Figure 3 illustrates the methodological quality of the trials and the possible risk of bias. Notably, all 10 of the included studies in this analysis share common features, such as a placebo-controlled design and double-blinded methodology, which contributes to the overall robustness of the research.

### 3.2 Details about the trials and the baseline characteristics of the participants

The baseline characteristics of subjects from all the RCTs included in the analytical and quantitative synthesis are delineated in both Tables 1, 2. The mean age of subjects varied across the trials, ranging from 14 years old in swimmers (35) to 29 years old in judo athletes (30). Likewise, there was considerable diversity in the daily dosage of vitamin D3 supplementation, with values ranging from a minimum of 2000 IU administered over 12 weeks (35) to a maximum of 18,750 IU delivered over 8 days (in the form of a bolus of 150,000 IU) (30).

**Table 1:** Characteristics of the included randomized controlled trials.

References	Country	Latitude	Season	Sports activity	Randomized (*n* = 354)	Intervention vitamin D dosage IU (μg)	Duration	Type	Product
Brzeziański et al. (34)	Poland	51.5° N	Winter	Soccer	12/13	40,000 (1000)/week vs. PL	8 weeks	Not reported	Decristol, Sun-Farm Sp. z o.o., Lomianki, Poland
Close et al. (29)	UK	53° N	Winter	Football and Rugby	10/10/10	20,000 (500)/week vs. 40,000 (1000)/week vs. PL	12 weeks	D_3_ capsules	Biotech Pharmacal Inc., Phoenix, AZ, USA
Close et al. (33)	UK	53° N	Winter	Soccer	5/5	5,000 (125)/day vs. PL	8 weeks	D_3_ capsules	Biotech Pharmacal Inc., Phoenix, AZ, USA
Dubnov-Raz et al. (35)	Israel	31.9–32.7° N	Winter	Swimming	27/26	2000 (50)/day vs. PL	12 weeks	D_3_ droplets	CTS Chemical Industries Ltd., Isreal
Fairbairn et al. (32)	New Zealand	45–46.5° S	Autumn	Rugby	28/29	3,570 IU (89 μg)/day	11–12 weeks	D_3_ tablets	Cal.D.Forte, PSM Healthcare, Auckland, New Zealand
Jastrzębska et al. (37)	Poland	50.1–54.5° N	Winter	Soccer	20/16	5,000 (125)/day vs. PL	8 weeks	D_3_ droplets	Vigantol, Merck, Germany
Jastrzębska et al. (36)	Poland	54.5° N	Winter	Soccer	18/17	5,000 (125)/day vs. PL	12 weeks	D_3_ droplets	Vigantol, Merck, Germany
Jung et al. (31)	Korea	33.3° N	Winter	Taekwondo	22/22	5,000 (125)/day vs. PL	4 weeks	D_3_ capsules	Bio-Tech Pharmacal, Inc. (Arkansas, USA)
Todd et al. (38)	UK	55° N	Winter	Soccer	22/20	3,000 (75)/day vs. PL	12 weeks	D_3_ solution	BetterYou, Ltd., Barnsley, UK
Wyon et al. (30)	UK	52.3° N	Winter	Judo	11/11	150,000 (3750)/one time vs. PL	8 days	D_3_ tablets	Not reported

Data are means unless stated otherwise; PL Placebo. 2013a, 30 football and rugby athletes were recruited in reference (29); 2013b, 10 soccer players were recruited in reference (33).

**Table 2:** Baseline measurements of the included randomized controlled trials.

References	Analyzed (*n* = 354)	VD3 daily dosage IU	Males (%)	Mean age years	25(OH)D (ng/mL)	25(OH)D (nmol/L)	25(OH)D method of analysis	Lost to follow-up
Brzeziański et al. (34)	12	5,714	100	17.5 ± 0.7	27.9 ± 10.79	69.75 ± 26.97	Elecsys Vitamin D Total II	0
	13	PL			25.5 ± 9.52	63.75 ± 23.8	(Roche Diagnostics GmbH, Germany)	
Close et al. (29)	10	5,714	100	21 ± 1	20.4 ± 10.4	51 ± 26	HPLC-MRM	5
	10	2,857		22 ± 2	21.2 ± 10.4	53 ± 26	(Becton Dickinson, UK)	−16.70%
	10	PL		21 ± 1	20.8 ± 10.8	52 ± 27		
Close et al. (33)	5	5,000	100	18 ± 5	11.6 ± 10	29 ± 25	HPLC-MRM (Becton Dickinson, Oxford, UK)	0
	5	PL			21.2 ± 11.6	53 ± 29		
Dubnov-Raz et al. (35)	27	2000	62% (33/53)	13.9 ± 1.6	24.4 ± 4.9	61 ± 12.25	RIA	6
	26	PL		14.1 ± 1.8	24.8 ± 4.6		(Diasorin, Minnesota, US)	−11.30%
Fairbairn et al. (32)	28	3,570	100	21.5 ± 2.8	37.2 ± 7.6	93 ± 19	LC–MS/MS at Canterbury Health	0
	29	PL		20.9 ± 2.8	38 ± 6.8	95 ± 17	New Zealand	
Jastrzębska et al. (37)	20	5,000	Not reported	17.5 ± 0.6	19.4 ± 3.44	48.5 ± 8.6	Not reported	0
	16	PL			19 ± 6.48	47.5 ± 16.2		
Jastrzębska et al. (36)	18	5,000	Not reported	17.2 ± 1.16	24.1 ± 5.23	60.25 ± 13.07	CMIA	9
	17	PL			24.8 ± 4.77	62 ± 11.92	(Liaison XL, DiaSorin, Saluggia, Italy)	−25.70%
Jung et al. (31)	22	5,000	60% (21/35)	20.1 ± 0.15	10.9 ± 2.35	27.3 ± 5.88	CLIA analyzer	9
	22	PL			12.4 ± 3.75	30.9 ± 9.38	(Liaison XL, Dasorin, Italy)	−20.45%
Todd et al. (38)	22	2000	23% (18/24)	20 ± 2	17.8 ± 5.3	44.49 ± 13.35	LCMS-MS	7
	20	PL		20 ± 2	16.4 ± 8.9	40.93 ± 22.09	(API 4000; AB SCIEX)	−16.67%
Wyon et al. (30)	11	18,750	100	29 ± 10.6	13.2 ± 3.8	32.8 ± 9.4	ECLIA	0
	11	PL		26 ± 7.4	16.3 ± 2.7	40.7 ± 6.8	(Tecan Infinite F500, Mannedorf, Switzerland)	

Data are mean ± standard deviation unless stated otherwise. CLIA, Chemiluminescent immunoassay; ECLIA, Electrochemiluminescent immunoassay; HPLC-MRM, High-performance liquid chromatography tandem multiple reaction mode; HPLC-MS, High-performance liquid chromatography–tandem mass spectrometry; LC–MS/MS, Liquid chromatography mass spectrometry; PL Placebo, RAI Radioimmunoassay. 2013a, 30 football and rugby athletes were recruited in reference (29); 2013b, 10 soccer players were recruited in reference (33).

In the study conducted by Wyon et al. (30), male Judo athletes were administered a solitary dose of 150,000 IU vitamin D3 tablets. The time interval between their post-intervention and pre-intervention assessments was 8 days. Close et al. conducted two distinct studies that included male athletes from soccer, football, and rugby players (29, 33). In one of the studies, participants were categorized into three groups and administered vitamin D3 capsules for a duration of 12 weeks, and the dosages used were 20,000 IU per week, 40,000 IU per week, and a placebo (29). In another investigation by Close et al. (33), male soccer players were given a daily dose of 5,000 IU vitamin D3 capsules for a period of 8 weeks. Jung et al. (31) carried out their study enrolling both male and female Taekwondo athletes for 4 weeks with 5,000 IU vitamin D3 supplementation on a daily basis. Fairbairn et al. (32) carried out an investigation involving male athletes who ingested 3,570 IU vitamin D3 tablets each day for a duration of 11 to 12 weeks. Brzeziański et al. (34) conducted an 8-week nutritional intervention administering a weekly dosage of 40,000 IU of vitamin D3 to male soccer players. Dubnov-Raz et al. (35) reported recruiting both male and female swimming athletes who were assigned a daily dosage of 2000 IU of vitamin D3 droplets for a duration of 12 weeks. Jastrzębska et al. (36, 37) carried out two studies, both focusing on the winter supplementation of vitamin D3 droplets in soccer players. One study (37) published in 2016 lasted for 8 weeks, while another study (36) published in 2022 had a duration of 12 weeks. Todd et al. (38) included male and female soccer athletes and provided them with a daily supplementation of 3,000 IU of vitamin D3 solution for a duration of 12 weeks.

### 3.3 Strength tests

**Figure 4:**
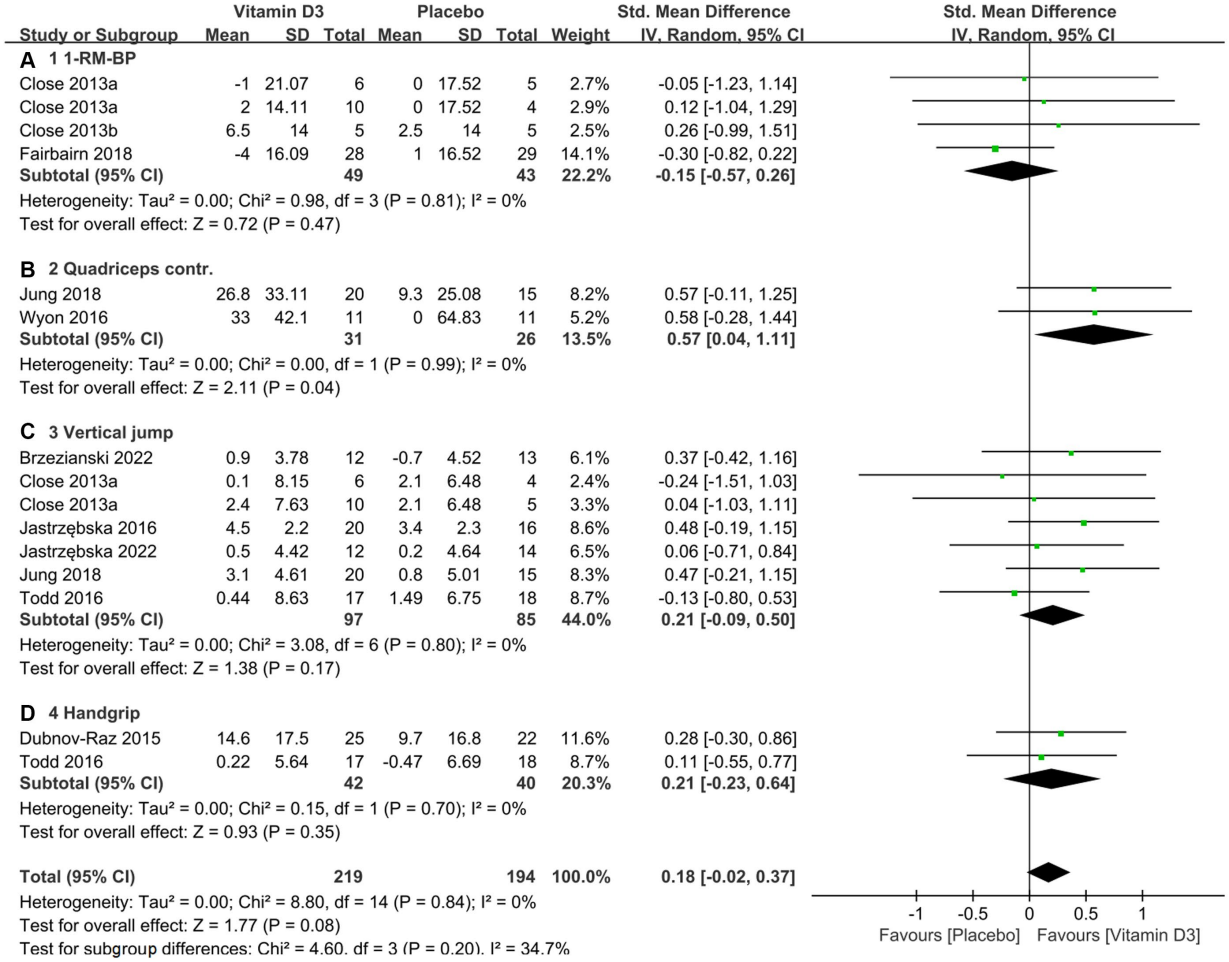
Forest plot for vitamin D3 supplementation effects on muscle strength. **(A)** 1-RM-BP; **(B)** Quadriceps contraction; **(C)** Vertical jump; **(D)** Handgrip strength test.

The total sample size for this study comprises 318 individuals (with a total of 554 observations), encompassing both the intervention and placebo groups. Table 3 offers a summary of the disparities in strength between vitamin D3 supplementation and placebo regarding one repetition maximum bench press (1-RM-BP) (also in Figure 4A), maximal quadriceps contraction (also in Figure 4B), vertical jump (CMJ) (also in Figure 4C), and handgrip (HG) strength (also in Figure 4D).

**Table 3:** Strength outcome measures.

References	Daily dosage	*N* = 318	1-RM BP (kg)			^a^Quadriceps Contr. (N·m)			Vertical jump (cm)			Handgrip (kg)		
	IU		Pre	Post	Change	Pre	Post	Change	Pre	Post	Change	Pre	Post	Change
Brzeziański et al. (34)	5,714	12							42.3 ± 3.88	43.2 ± 3.67	0.9 ± 3.78			
	0	13							45.4 ± 4.8	44.7 ± 4.17	−0.7 ± 4.52			
Close et al. (29)	5,714	6	91 ± 22	90 ± 20	−1 ± 21.07				47.1 ± 6.7	49.5 ± 8.3	2.4 ± 7.63			
	2,857	10	90 ± 13	92 ± 15	2 ± 14.11				49.1 ± 7.1	49.2 ± 8.9	0.1 ± 8.15			
	0	9	79 ± 17	79 ± 18	0 ± 17.52				45.6 ± 6.8	47.7 ± 6.1	2.1 ± 6.48			
^b^Close et al. (33)	5,000	5	82 ± 14	88.5 ± 14	6.5 ± 14									
	0	5	82 ± 14	84.5 ± 14	2.5 ± 14									
Dubnov-Raz et al. (35)	2000	25										120 ± 48	139 ± 50	14.6 ± 17.5
	0	22										109 ± 42	117 ± 45	9.7 ± 16.8
Fairbairn et al. (32)	3,570	28	126 ± 17	122 ± 15	−4 ± 16.09									
	0	29	122 ± 17	123 ± 16	1 ± 16.52									
Jastrzębska et al. (37)	5,000	20							41.2 ± 5.2	45.7 ± 5.2	4.5 ± 2.3			
	0	16							43 ± 4.9	46.4 ± 4.9	3.4 ± 2.2			
Jastrzębska et al. (36)	5,000	12							43.7 ± 4.91	44.2 ± 3.66	0.5 ± 4.42			
	0	14							44.7 ± 4.95	44.9 ± 4.26	0.2 ± 4.64			
Jung et al. (31)	5,000	20				323.6 ± 32.7 (60°)	350.4 ± 33.5	26.8 ± 33.11	54.1 ± 4.61	57.2 ± 4.61	3.1 ± 4.61			
	0	15				329.9 ± 24.4 (60°)	339.2 ± 25.7	9.3 ± 25.08	53.9 ± 5.01	54.7 ± 5.01	0.8 ± 5.01			
						3502.2 ± 790.02 (180°)	3782.1 ± 878.12	280 ± 837.55						
						3576.9 ± 980.36 (180°)	3549.4 ± 980.36	−27.5 ± 980.36						
Todd et al. (38)	2000	17							31.71 ± 8.32	32.15 ± 8.91	0.44 ± 8.63	36.78 ± 10.04	36.77 ± 11.05	0.18 ± 5.64
	0	18							27.36 ± 6.49	28.85 ± 6.99	1.49 ± 6.75	29.71 ± 11.56 (left)	32.98 ± 11.30	3.56 ± 6.69
												39.29 ± 10.57	39.51 ± 10.76	−0.03 ± 5.51
												30.54 ± 11.33 (right)	30.07 ± 11.30	3.3 ± 5.98
Wyon et al. (30)	18,750	11				232 ± 37.4 (30°)	265 ± 45.6	33 ± 42.1						
	0	11				239 ± 65.9 (30°)	239 ± 63.7	0 ± 64.83						
						163 ± 28.25 (200°)	183 ± 32.97	20 ± 30.88						
						145 ± 29.27 (200°)	148 ± 28.21	3 ± 28.75						

^a^Did maximal quadriceps contraction at 30°/s, 60°/s, 180°/s and 200°/s; ^b^Baseline measured from an average from 10 participants.

Figure 4 forest plots were created to present the results of various indicators based on different strength tests. In accordance with the available evidence, our study’s findings suggested that there were no substantial improvements observed in 1-RM-BP (SMD −0.15, 95% CI: −0.57 to 0.26, *p* = 0.47), vertical jump (SMD 0.21, 95% CI: −0.09 to 0.50, *p* = 0.17), and handgrip strength (SMD 0.21, 95% CI: −0.23 to 0.64, *p* = 0.35). However, there was significant enhancement in maximal quadriceps contraction (SMD 0.57, 95% CI: 0.04 to 1.11, *p* = 0.04). Meanwhile, the pooled analysis suggested that the overall strength tests demonstrated no significant improvement (SMD 0.18, 95% CI: −0.02 to 0.37, *p* = 0.08).

### 3.4 Concentrations of serum 25(OH)D

#### 3.4.1 The influence of vitamin D3 supplementation on the status of serum 25(OH)D in the included research studies

**Figure 5:**
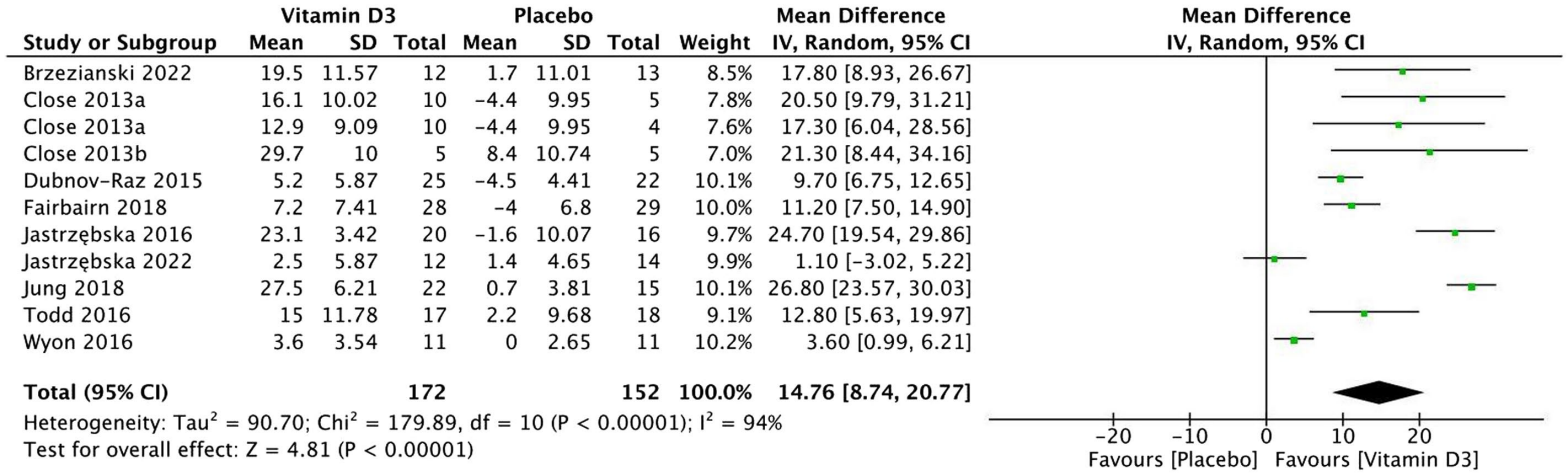
Forest plot for vitamin D3 supplementation effects on serum 25(OH)D status.

Tables 4, 5 present a comprehensive summary of the average serum 25(OH)D concentrations at the baseline and the post-intervention of each study. Remarkably, in cases where athletes initially had serum 25(OH)D levels below 25 ng/mL, the administration of vitamin D3 supplements (at doses varying from 2000 to 18,750 IU/day and durations ranging from 1 to 12 weeks) has been shown to improve their vitamin D status. To be more precise, Fairbairn (32) and Brzezianski (34) noted that athletes with initially adequate vitamin D status exhibited a rise in serum 25(OH)D levels when given daily doses of 3,570 IU and 5,714 IU, respectively, in contrast to the placebo group (see Table 4). Additionally, it is worth mentioning that in their study, Wyon (30) provided their participants with a single bolus of 150,000 IU of vitamin D3 supplementation. Even though the average serum 25(OH)D levels stayed below 30 ng/mL on the eighth day after the administration of the dose, there was an enhancement in the changes of serum 25(OH)D status when compared to the placebo group. In comparison with the placebo group, there is a significant increase in serum 25(OH)D levels among athletes following a period of vitamin D3 supplementation (MD 14.76, 95% CI: 8.74 to 20.77, *p* < 0.0001). The details of serum 25(OH)D changes were illustrated in Figure 5.

**Table 4:** Baseline and follow-up Serum 25(OH)D concentrations.

References	Latitude	Time	Vitamin D3 daily dosage IU	Baseline (ng/mL)	*N* = 318	1 Week (ng/mL)	4 Weeks (ng/mL)	6 Weeks (ng/mL)	8 Weeks (ng/mL)	12 Weeks (ng/mL)
Brzeziański et al. (34)	51.5° N	Jan–Mar	5,714	27.9 ± 10.79	12				47.4 ± 12.21	
			0	25.5 ± 9.52	13				27.2 ± 12.06	
Close et al. (29)	53° N	Jan–Apr	5,714	20.4 ± 10.4	6			39.3 ± 5.6		36.5 ± 9.6
			2,857	21.2 ± 10.4	10			31.7 ± 5.6		34.1 ± 4.0
			0	20.8 ± 10.8	9			14.8 ± 7.2		16.4 ± 8.8
Close et al. (33)	53° N	Nov–Jan	5,000	11.6 ± 10.0	5				41.3 ± 10.0	
			0	21.2 ± 11.6	5				29.6 ± 9.6	
Dubnov-Raz et al. (35)	31.9–32.7° N	Nov–Jan	2000	24.4 ± 4.9	25					29.6 ± 6.5
			0	24.8 ± 4.6	22					20.3 ± 4.2
Fairbairn et al. (32)	45–46.5° S	Mar–May	3,570	37.2 ± 7.6	28			44.4 ± 7.2		45.6 ± 7.6
			0	38 ± 6.8	29			34 ± 6.8		32 ± 8.4
Jastrzębska et al. (37)	50.1–54.5° N	Jan–Mar	5,000	19.4 ± 3.44	20				42.52 ± 3.4	
			0	19 ± 6.48	16				17.4 ± 11.6	
Jastrzębska et al. (36)	54.5° N	Jan–Mar	5,000	24.1 ± 5.23	12					26.6 ± 6.35
			0	24.8 ± 4.77	14					26.2 ± 4.52
Jung et al. (31)	33.3° N	Jan–Feb	5,000	10.9 ± 2.35	20		38.4 ± 7.04			
			0	12.4 ± 3.75	15		13.1 ± 3.87			
Todd et al. (38)	55° N	Nov–Apr	2000	17.8 ± 5.3	17					32.8 ± 13.5
			0	16.4 ± 8.9	18					18.6 ± 10.3
Wyon et al. (30)	52.3° N	Feb	18,750	13.2 ± 3.8	11	16.8 ± 3.2				
			0	16.3 ± 2.7	11	16.3 ± 2.6				

Data are mean ± standard deviation unless stated otherwise. Measurements are in ng/mL.

**Table 5:** Baseline and end-point mean 25(OH)D concentrations in vitamin D and placebo.

References	Latitude	Vitamin D daily dosage (IU)	Weeks	Vitamin D Supplementation^a^				Placebo^a^			
				N^c^	Pre	Post	Change	N^c^	Pre	Post	Change
Vitamin D < 25 ng/mL (*N*^c^ = 272/236)											
Winter (*N*^c^ = 297/261)											
^b^Close et al. (29)	≥45° N	5,714	12	10/6	20.4 ± 10.4	36.5 ± 9.6	16.1 ± 10.02	10^/9^	20.8 ± 10.8	16.4 ± 8.8	−4.4 ± 9.95
^b^Close et al. (29)	≥45° N	2,857	12	10	21.2 ± 10.4	34.1 ± 4.0	12.9 ± 9.09	10^/9^	20.8 ± 10.8	16.4 ± 8.8	−4.4 ± 9.95
Close et al. (33)	≥45° N	5,000	6	5	11.6 ± 10.0	41.3 ± 10.0	29.7 ± 10	5	21.2 ± 11.6	29.6 ± 9.6	8.4 ± 10.74
Dubnov-Raz et al. (35)	<45° N	2000	12	27/25	24.4 ± 4.9	29.6 ± 6.5	5.2 ± 5.87	26/22	24.8 ± 4.6	20.3 ± 4.2	−4.5 ± 4.41
Jastrzębska et al. (37)	≥45° N	5,000	8	20	19.4 ± 3.44	42.5 ± 3.4	23.1 ± 3.42	16	19 ± 6.48	17.4 ± 11.6	−1.6 ± 10.07
Jastrzębska et al. (36)	≥45° N	5,000	12	18/12	24.1 ± 5.23	26.6 ± 6.35	2.5 ± 5.87	17/14	24.8 ± 4.77	26.2 ± 4.52	1.4 ± 4.65
Jung et al. (31)	<45° N	5,000	4	22	10.9 ± 2.35	38.4 ± 7.04	27.5 ± 6.21	22/15	12.4 ± 3.75	13.1 ± 3.87	0.7 ± 3.81
Todd et al. (38)	≥45° N	3,000	12	22/17	17.8 ± 5.3	32.8 ± 13.5	15 ± 12.94	20/18	16.4 ± 8.9	18.6 ± 10.3	2.44 ± 9.57
Wyon et al. (30)	≥45° N	18,750	1	11	13.2 ± 3.8	16.8 ± 3.2	3.6 ± 3.54	11	16.3 ± 2.7	16.3 ± 2.6	0 ± 2.65
Vitamin D > 25 ng/mL (*N* = 82)											
Autumn (*N* = 57)											
Brzezianski et al. (34)	≥45° N	5,714	8	12	27.9 ± 10.79	47.4 ± 12.21	19.5 ± 11.57	13	25.5 ± 9.52	27.2 ± 12.06	1.7 ± 11.01
Fairbairn et al. (32)	≥45° S	3,570	12	28	37.2 ± 7.6	44.4 ± 7.2	7.2 ± 7.41	29	38 ± 6.8	34 ± 6.8	−4 ± 6.8

^a^Presented in ng/mL. ^b^Close et al. compared multiple doses with one control group. ^c^Number of baseline/post-intervention. ^ Indicates findings are from one study.

#### 3.4.2 An analysis of sensitivity regarding the impact of vitamin D3 supplementation on serum 25(OH)D status

A sensitivity analysis was performed by excluding trials in which attrition, defined as the loss of more than 15% of participants between the initial assessment and the study’s conclusion, occurred. Four studies [Close et al. (29) −16.7%, Jastrzębska et al. (36) −25.7%, Jung et al. (31) −20.45%, Todd et al. (38) −16.67%] reported attrition rates exceeding 15% of participants at the conclusion of the study. However, systematically removing these four studies does not have a significantly impact on the overall findings (see Supplementary material). The evidence continues to endorse the overall positive impact of vitamin D3 supplementation on serum 25(OH)D levels.

## 4 Discussion

### 4.1 Main findings overview

Based on the results from our updated meta-analysis, we can infer that vitamin D3 supplementation, when administered for a duration of 4–12 weeks at a daily dose surpassing 2000 IU, is significantly effective in elevating athletes’ serum 25(OH)D concentrations from insufficient to sufficient levels, especially during the winter season. The advantageous effects of vitamin D3 supplementation on muscle strength were demonstrated majorly through quadriceps strength, however, the overall effect sizes for combining vertical jump, quadriceps contraction, vertical jump, and 1-RM-BP did not show a significant improvement.

#### 4.1.1 Muscle strength

In order to ensure consistency in the pooled mean difference between the vitamin D3 supplementation and placebo groups, each subgroup included two or more observations that contributed to the aggregation of SMDs for strength measurements. Although there were no significant improvements observed in 1-RM BP (SMD 0.15, 95% CI: −0.57 to 0.26, *p* = 0.47), handgrip strength (SMD 0.21, 95% CI: −0.23 to 0.64, *p* = 0.35), and vertical jump (SMD 0.21, 95% CI: −0.09 to 0.50, *p* = 0.17), a significant enhancement was observed in maximal quadriceps contraction (SMD 0.57, 95% CI: 0.04 to 1.11, *p* = 0.04). It is noteworthy that with increased sample size from our previous meta-analysis accomplished in 2019, there is still no significant increase been observed in the overall strength test (SMD 0.18, 95% CI: −0.02 to 0.37, *p* = 0.08) combining the four indicators mentioned above.

Therefore, the advantageous effects of vitamin D3 supplementation on muscle strength were demonstrated through quadriceps strength. However, the overall effect sizes for combining vertical jump, handgrip, quadriceps contraction, and 1-RM-BP did not show a significant improvement. Additionally, it is important to note that the quantitative synthesis of either the handgrip test or quadriceps contraction test was conducted based on a limited number of subjects from only two included studies. This limitation highlights the need for future meta-analyses to further investigate this aspect.

#### 4.1.2 Serum 25(OH)D levels

The results depicted in Figure 5 suggests that vitamin D3 supplementation yields a favorable effect on the average serum 25(OH)D concentrations compared to placebo supplementation. Furthermore, the administration of 5,000 IU of vitamin D3 for a duration of 4 weeks successfully increased the serum 25(OH)D levels of participants from a state of deficiency to sufficiency, particularly at a latitude of 33.3° N during the winter (31). In conclusion, there exists a substantial body of evidence consistently supporting the advantageous impact of vitamin D3 supplementation on serum 25(OH)D levels in comparison to placebo supplementation.

### 4.2 Strengths and weaknesses

The study adhered to the PRISMA criteria, which are commonly applied in Cochrane reviews, to guarantee the quality and rigor of the methodology. The selection and review processes were carried out independently by two reviewers, thereby enhancing the reliability of the study’s conclusions. In addition, it should be emphasized that the study’s conclusions are grounded in the latest and officially published RCTs that are available, further strengthening the credibility and rigor of this systematic review and meta-analysis.

Although this updated meta-analysis includes two additional measures to evaluate muscle strength compared to the 2019 quantitative synthesis, it is important to acknowledge the limited availability of data for certain evaluation measures, such as grip strength and quadriceps contraction. It’s important to acknowledge that this study incorporates various supplementation dosages, outcome measurements, sports, and training routines, which may introduce potential confounding variables and heterogeneity.

It is regrettable that our study possesses inherent limitations commonly associated with systematic reviews and meta-analyses, and it is essential to acknowledge and consider these limitations. For example, sports that involve year-round indoor training, such as Judo and Taekwondo, may potentially result in a notable decrease in serum 25(OH)D levels when compared to outdoor sports. Furthermore, the importance of muscle strength can fluctuate among various sports, with certain athletes, such as those in Taekwondo and Judo, prioritizing strength enhancement to a higher degree compared to athletes in sports like soccer. These factors introduce complexity and potential variability into the study’s findings. Due to the limited number of RCTs available for examining the effects of vitamin D3 supplementation on muscle strength, it becomes challenging to account for various variables and factors that might influence the outcomes. This scarcity of RCTs can limit the ability to conduct more detailed and comprehensive analyses that consider multiple factors and potential confounders. These variables could include the timing of measurements during different seasons of the year, the specific characteristics of sports professionals, sunlight exposure, specific age groups, gender differences, dietary factors (such as Mediterranean diet, vegan diet, Ketogenic diet), and more. These complexities make it challenging to account for all potential confounding factors in our analysis.

Our study reveals that vitamin D3 has presented positive impact on muscle strength, particularly evident in the quadriceps strength. Indeed, the study recognizes the relatively modest sample size and the difficulties associated with stratifying athletes to achieve improved control during outcome aggregation and summarize. This suggests that elevating serum 25(OH)D concentrations can be achieved with the correct dosage and duration of vitamin D supplementation, and there is some evidence indicating an enhancement in muscle strength as a consequence.

It is important to emphasize that the trials included in this analysis is indeed featured with small sample sizes, spanning from 10 to 57 participants, and there was a discernible disparity in baseline serum 25(OH)D levels among the studies (29–38). Furthermore, this study included individuals from various sports professions with diverse nationalities, living in different latitudes, and in one case, a study with only 5 athletes who received vitamin D3 intervention (33). These factors contribute to the complexity and heterogeneity of the data, and they should be considered when interpreting the results. These factors contribute to the overall complexity and heterogeneity of the data. Nevertheless, the RCTs included in our analysis exhibit a high level of consistency in these aspects. It is noteworthy that 70% of the trials were conducted in higher latitude regions such as the UK and Poland. Moreover, apart from the research conducted by Fairbairn (32), the remaining nine studies were carried out during the winter season, which is characterized by reduced sunlight exposure. Furthermore, it is worth mentioning that 70% of the studies included participants who were outdoor athletes engaged in various sports, such as soccer and rugby. This variation in the types of sports and outdoor activities may also introduce additional complexity and diversity into the study population.

Sunlight exposure plays a pivotal role in the synthesis of vitamin D in the human body (39). Athletes who follow weight management protocols (40) and experience restricted sunlight exposure, as observed in sports like figure skating (41) and ice hockey (42–44), are recognized for having an elevated incidence of vitamin D deficiency. This highlights the significance of obtaining adequate sunlight exposure to sustain sufficient vitamin D levels, particularly for individuals involved in indoor or cold-weather sports. Based on the aforementioned evidence, future research should explore more comprehensive outcomes of vitamin D3 supplementation, thereby enhancing the practical relevance of future meta-analysis results.

The selected RCTs revealed a range of findings concerning the influence of vitamin D supplementation. In particular, nine studies (45–53) indicated that vitamin D supplementation successfully raised serum 25(OH)D levels, although they did not establish a definitive connection between vitamin D3 supplementation and muscle strength. Interestingly, three studies (49–51) demonstrated that vitamin D2 supplementation notably increased serum 25(OH)D2 levels but decreased serum 25(OH)D3 levels, resulting in no significant impacts on strength assessments. In mouse models that were exclusively provided with either vitamin D2 or vitamin D3 in their diet, it was observed that by week 16, vitamin D2-fed mice exhibited improved bone health in comparison to vitamin D3-fed mice (54). However, different researchers have reported that vitamin D2 supplementation is less efficient than vitamin D3 in sustaining optimal serum 25(OH)D levels (55–59). Moreover, findings from the Longitudinal Aging Study Amsterdam (60) suggest that lower vitamin D levels and increased parathyroid hormone levels may act as indicators of muscle strength decline. These divergent findings emphasize the intricate and multifaceted nature of the interplay between vitamin D supplementation, serum levels, and muscle strength.

Indeed, there are many reports indicating that vitamin D3 supplementation can improve physical fitness (60–63), and our updated meta-analysis conducted on high-quality RCTs aimed to provide substantial evidence support this rationale specifically focusing on vitamin D3 supplementation for athletes. Unfortunately, with increased sample size for quantitative meta-analysis, there is still no overall significant effect (*p* = 0.08) been observed compared to no intervention control group.

### 4.3 Implications for further research and practical applications

This meta-analysis investigates the recent effects of vitamin D3 supplementation on serum 25(OH)D levels and muscle strength, using data obtained from randomized controlled trials. It serves as an extension of a previous systematic review and meta-analysis conducted in 2019. This supplementation regimen aims to maintain sufficient serum 25(OH)D concentrations, thereby enhancing muscle strength and improving athletic performance. The advantageous effects of vitamin D3 supplementation on muscle strength, particularly through quadriceps strength, lacks sufficient support from the results. The present analysis, while suggestive of a potential trend, falls short of providing conclusive evidence. Moreover, the uncertainty is further heightened by the limited number of studies specifically examining quadriceps strength, alongside the variability observed in studies analyzing 1-RM-BP and Vertical Jump. These factors underscore the necessity for additional research to establish firmer conclusions.

## 5 Conclusion

Although, potential benefit of vitamin D supplementation for enhancing muscle strength was found in athletes with limited available studies for the quantitative synthesis, it cannot warrant significant overall enhancements in muscle strength when athletes attain adequate serum 25(OH)D levels through supplementation.

## Data availability statement

The original contributions presented in the study are included in the article/Supplementary material, further inquiries can be directed to the corresponding author.

## Author contributions

QH: Writing – original draft. MX: Formal analysis, Methodology, Software, Validation, Visualization, Writing – original draft. NA: Funding acquisition, Project administration, Supervision, Writing – review & editing. QT: Formal analysis, Writing – review & editing. JS: Formal analysis, Writing – original draft. QW: Funding acquisition, Project administration, Supervision, Writing – review & editing.
