# Supplementary material for: Effects of vitamin D3 supplementation on strength of lower and upper extremities in athletes: an updated systematic review and meta-analysis of randomized controlled trials
Source: Front Nutr. 2024 May 27;11:1381301. doi: 10.3389/fnut.2024.1381301 (PMC11163122; doi:10.3389/fnut.2024.1381301)
Supplement: Supplementary file 1 [file Image_1.pdf]

Search Methods for Identification of Articles

An electronic literature search of the PubMed, EBSCO, and Cochrane Library databases from inception to September 27, 2023 was completed. Combinations of the following search terms and medical subject headings (MeSH) were used: vitamin D, 25-hydroxyvitamin D, 25(OH)D, cholecalciferol, calcidiol, 25-hydroxycholecalciferol, exercise, athletes, sports, athletic performance, physical fitness. The search results were merged and duplicates were removed and the searching strategy was as follows:

(("vitamin d"[MeSH Terms] OR "vitamin d"[All Fields] OR "ergocalciferols"[MeSH Terms] OR "ergocalciferols"[All Fields]) OR ("25-hydroxyvitamin D"[Supplementary Concept] OR "25-hydroxyvitamin D"[All Fields] OR "25 hydroxyvitamin d"[All Fields] OR "calcifediol"[MeSH Terms] OR "calcifediol"[All Fields]) OR 25[All Fields] AND ("hydroxide ion"[Supplementary Concept] OR "hydroxide ion"[All Fields] OR "oh"[All Fields]) AND D[All Fields] OR ("cholecalciferol"[MeSH Terms] OR "cholecalciferol"[All Fields]) OR ("calcifediol"[MeSH Terms] OR "calcifediol"[All Fields] OR "calcidiol"[All Fields]) OR ("calcifediol"[MeSH Terms] OR "calcifediol"[All Fields] OR "25 hydroxycholecalciferol"[All Fields])) AND (("exercise"[MeSH Terms] OR "exercise"[All Fields]) OR ("athletes"[MeSH Terms] OR "athletes"[All Fields] OR "athlete"[All Fields]) OR ("sports"[MeSH Terms] OR "sports"[All Fields] OR "athletic"[All Fields]) OR ("sports"[MeSH Terms] OR "sports"[All Fields]) OR ("physical fitness"[MeSH Terms] OR ("physical"[All Fields] AND "fitness"[All Fields]) OR "physical fitness"[All Fields]))

	Pre-Vitamin D3			Post-Vitamin D3				Mean Difference
Study or Subgroup	Mean [ng/ml]	SD [ng/ml]	Total	Mean [ng/ml]	SD [ng/ml]	Total	Weight	IV, Random, 95% CI
Brzezianski 2022	27.9	10.79	13	47.4	12.21	13	9.6%	−19.50 [−28.36, −10.64]
Close 2013a	21.2	10.4	10	34.1	4	10	0.0%	−12.90 [−19.81, −5.99]
Close 2013a	20.4	10.4	6	36.5	9.6	6	8.7%	−16.10 [−27.42, −4.78]
Close 2013b	11.61	10	5	41.3	10	5	8.3%	−29.69 [−42.09, −17.29]
Dubnov-Raz 2015	24.8	4.6	26	29.6	6.5	26	11.1%	−4.80 [−7.86, −1.74]
Fairbairn 2018	37.2	7.6	28	44.4	7.2	28	10.9%	−7.20 [−11.08, −3.32]
Jastrzębska 2016	19.4	3.4	20	42.52	3.4	20	11.2%	−23.12 [−25.23, −21.01]
Jastrzębska 2022	24.5	6.26	11	40.5	6.86	11	10.6%	−16.00 [−21.49, −10.51]
Jung 2018	10.9	2.35	20	38.4	7.04	20	11.0%	−27.50 [−30.75, −24.25]
Todd 2016	47.37	13.29	22	83.68	32.98	22	7.5%	−36.31 [−51.17, −21.45]
Wyon 2016	13.2	3.8	11	16.8	3.2	11	11.1%	−3.60 [−6.54, −0.66]
**Total (95% CI)**			**162**			**162**	**100.0%**	**−17.50 [−24.51, −10.49]**

Heterogeneity: Tau^2 = 113.13; Chi^2 = 243.56, df = 9 (P < 0.00001); I^2 = 96%
Test for overall effect: Z = 4.89 (P < 0.00001)

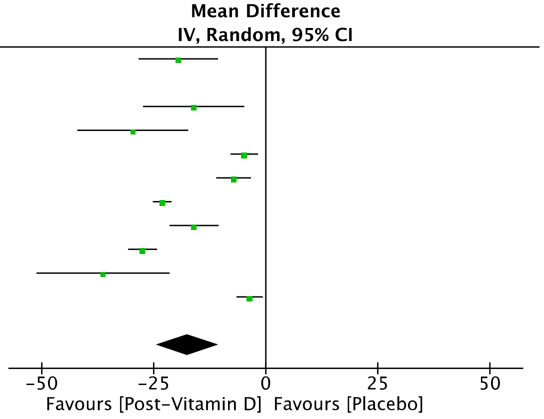

(A)

	Pre-Vitamin D3			Post-Vitamin D3				Mean Difference
Study or Subgroup	Mean [ng/ml]	SD [ng/ml]	Total	Mean [ng/ml]	SD [ng/ml]	Total	Weight	IV, Random, 95% CI
Brzezianski 2022	27.9	10.79	13	47.4	12.21	13	9.4%	−19.50 [−28.36, −10.64]
Close 2013a	21.2	10.4	10	34.1	4	10	10.0%	−12.90 [−19.81, −5.99]
Close 2013a	20.4	10.4	6	36.5	9.6	6	0.0%	−16.10 [−27.42, −4.78]
Close 2013b	11.61	10	5	41.3	10	5	8.2%	−29.69 [−42.09, −17.29]
Dubnov-Raz 2015	24.8	4.6	26	29.6	6.5	26	10.9%	−4.80 [−7.86, −1.74]
Fairbairn 2018	37.2	7.6	28	44.4	7.2	28	10.8%	−7.20 [−11.08, −3.32]
Jastrzębska 2016	19.4	3.4	20	42.52	3.4	20	11.0%	−23.12 [−25.23, −21.01]
Jastrzębska 2022	24.5	6.26	11	40.5	6.86	11	10.4%	−16.00 [−21.49, −10.51]
Jung 2018	10.9	2.35	20	38.4	7.04	20	10.9%	−27.50 [−30.75, −24.25]
Todd 2016	47.37	13.29	22	83.68	32.98	22	7.3%	−36.31 [−51.17, −21.45]
Wyon 2016	13.2	3.8	11	16.8	3.2	11	10.9%	−3.60 [−6.54, −0.66]
**Total (95% CI)**			**166**			**166**	**100.0%**	**−17.14 [−24.02, −10.26]**

Heterogeneity: Tau^2 = 110.29; Chi^2 = 244.10, df = 9 (P < 0.00001); I^2 = 96%
Test for overall effect: Z = 4.88 (P < 0.00001)

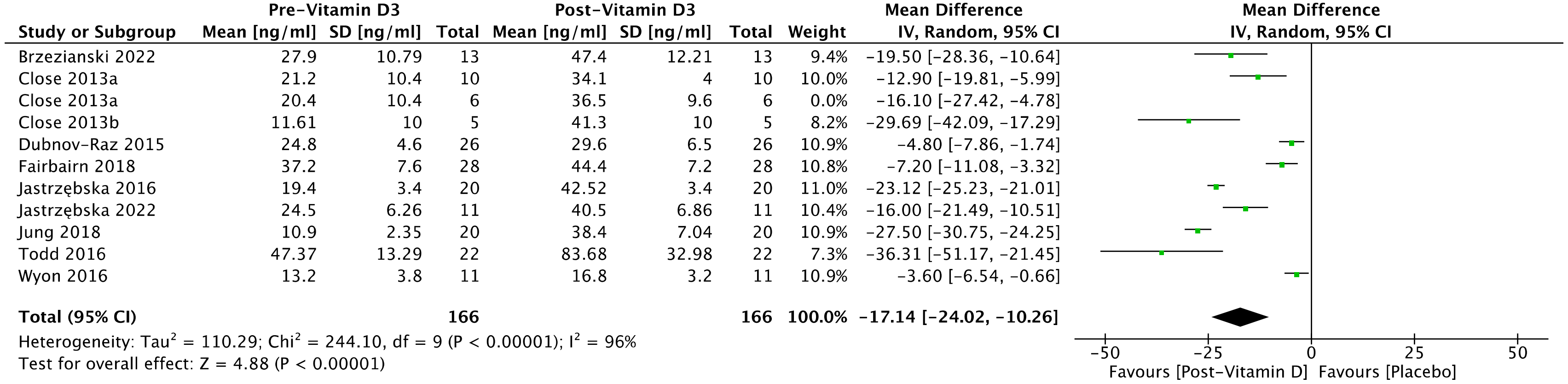

(B)

Study or Subgroup	Pre-Vitamin D3 Mean [ng/ml]	Pre-Vitamin D3 SD [ng/ml]	Pre-Vitamin D3 Total	Post-Vitamin D3 Mean [ng/ml]	Post-Vitamin D3 SD [ng/ml]	Post-Vitamin D3 Total	Weight	Mean Difference IV, Random, 95% CI
Brzezianski 2022	27.9	10.79	13	47.4	12.21	13	9.7%	−19.50 [−28.36, −10.64]
Close 2013a	21.2	10.4	10	34.1	4	10	10.3%	−12.90 [−19.81, −5.99]
Close 2013a	20.4	10.4	6	36.5	9.6	6	8.8%	−16.10 [−27.42, −4.78]
Close 2013b	11.61	10	5	41.3	10	5	8.4%	−29.69 [−42.09, −17.29]
Dubnov-Raz 2015	24.8	4.6	26	29.6	6.5	26	11.2%	−4.80 [−7.86, −1.74]
Fairbairn 2018	37.2	7.6	28	44.4	7.2	28	11.1%	−7.20 [−11.08, −3.32]
Jastrzębska 2016	19.4	3.4	20	42.52	3.4	20	0.0%	−23.12 [−25.23, −21.01]
Jastrzębska 2022	24.5	6.26	11	40.5	6.86	11	10.7%	−16.00 [−21.49, −10.51]
Jung 2018	10.9	2.35	20	38.4	7.04	20	11.2%	−27.50 [−30.75, −24.25]
Todd 2016	47.37	13.29	22	83.68	32.98	22	7.5%	−36.31 [−51.17, −21.45]
Wyon 2016	13.2	3.8	11	16.8	3.2	11	11.2%	−3.60 [−6.54, −0.66]
**Total (95% CI)**			**152**			**152**	**100.0%**	**−16.36 [−23.28, −9.45]**

Heterogeneity: $Tau^2 = 108.54$; $Chi^2 = 169.22$, df = 9 (P < 0.00001); $I^2 = 95\%$
Test for overall effect: Z = 4.64 (P < 0.00001)

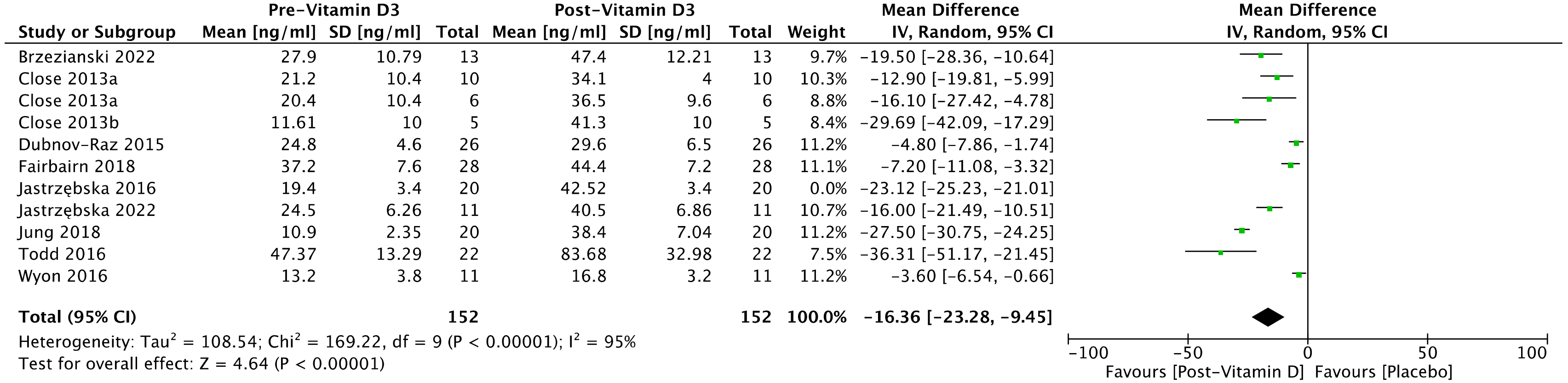

(C)

Study or Subgroup	Pre-Vitamin D3 Mean [ng/ml]	Pre-Vitamin D3 SD [ng/ml]	Pre-Vitamin D3 Total	Post-Vitamin D3 Mean [ng/ml]	Post-Vitamin D3 SD [ng/ml]	Post-Vitamin D3 Total	Weight	Mean Difference IV, Random, 95% CI
Brzezianski 2022	27.9	10.79	13	47.4	12.21	13	9.6%	−19.50 [−28.36, −10.64]
Close 2013a	21.2	10.4	10	34.1	4	10	10.3%	−12.90 [−19.81, −5.99]
Close 2013a	20.4	10.4	6	36.5	9.6	6	8.6%	−16.10 [−27.42, −4.78]
Close 2013b	11.61	10	5	41.3	10	5	8.2%	−29.69 [−42.09, −17.29]
Dubnov-Raz 2015	24.8	4.6	26	29.6	6.5	26	11.3%	−4.80 [−7.86, −1.74]
Fairbairn 2018	37.2	7.6	28	44.4	7.2	28	11.1%	−7.20 [−11.08, −3.32]
Jastrzębska 2016	19.4	3.4	20	42.52	3.4	20	11.5%	−23.12 [−25.23, −21.01]
Jastrzębska 2022	24.5	6.26	11	40.5	6.86	11	10.7%	−16.00 [−21.49, −10.51]
Jung 2018	10.9	2.35	20	38.4	7.04	20	0.0%	−27.50 [−30.75, −24.25]
Todd 2016	47.37	13.29	22	83.68	32.98	22	7.3%	−36.31 [−51.17, −21.45]
Wyon 2016	13.2	3.8	11	16.8	3.2	11	11.3%	−3.60 [−6.54, −0.66]
**Total (95% CI)**			**152**			**152**	**100.0%**	**−15.80 [−22.40, −9.20]**

Heterogeneity: $Tau^2 = 97.81$; $Chi^2 = 183.30$, df = 9 (P < 0.00001); $I^2 = 95\%$
Test for overall effect: Z = 4.69 (P < 0.00001)

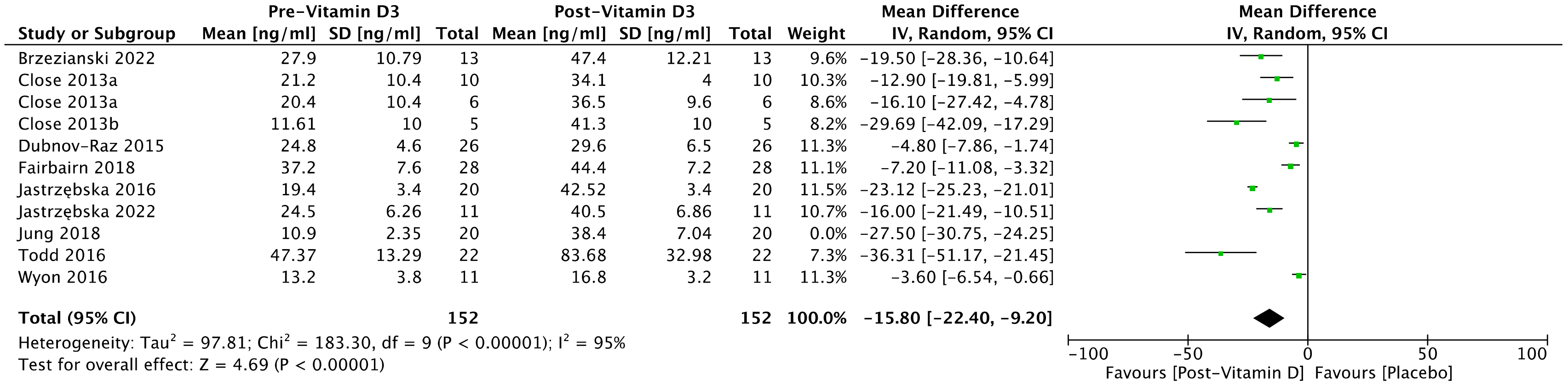

(D)

	Pre-Vitamin D3			Post-Vitamin D3				Mean Difference
**Study or Subgroup**	**Mean [ng/ml]**	**SD [ng/ml]**	**Total**	**Mean [ng/ml]**	**SD [ng/ml]**	**Total**	**Weight**	**IV, Random, 95% CI**
Brzezianski 2022	27.9	10.79	13	47.4	12.21	13	9.3%	−19.50 [−28.36, −10.64]
Close 2013a	21.2	10.4	10	34.1	4	10	9.9%	−12.90 [−19.81, −5.99]
Close 2013a	20.4	10.4	6	36.5	9.6	6	8.4%	−16.10 [−27.42, −4.78]
Close 2013b	11.61	10	5	41.3	10	5	8.0%	−29.69 [−42.09, −17.29]
Dubnov-Raz 2015	24.8	4.6	26	29.6	6.5	26	10.8%	−4.80 [−7.86, −1.74]
Fairbairn 2018	37.2	7.6	28	44.4	7.2	28	10.7%	−7.20 [−11.08, −3.32]
Jastrzębska 2016	19.4	3.4	20	42.52	3.4	20	10.9%	−23.12 [−25.23, −21.01]
Jastrzębska 2022	24.5	6.26	11	40.5	6.86	11	10.3%	−16.00 [−21.49, −10.51]
Jung 2018	10.9	2.35	20	38.4	7.04	20	10.8%	−27.50 [−30.75, −24.25]
Todd 2016	47.37	13.29	22	83.68	32.98	22	0.0%	−36.31 [−51.17, −21.45]
Wyon 2016	13.2	3.8	11	16.8	3.2	11	10.8%	−3.60 [−6.54, −0.66]
**Total (95% CI)**			**150**			**150**	**100.0%**	**−15.65 [−22.36, −8.94]**

Heterogeneity: $Tau^2 = 106.00$; $Chi^2 = 236.50$, df = 9 ($P < 0.00001$); $I^2 = 96\%$

Test for overall effect: Z = 4.57 ($P < 0.00001$)

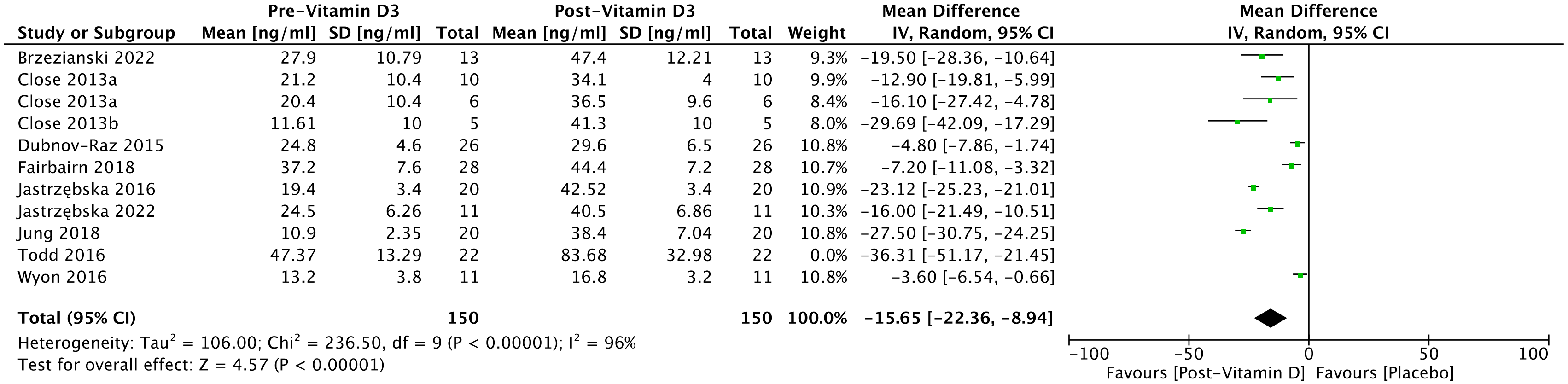

(E)

Supplemental Figure 1 Forests plot displayed a meta-analysis examining the impact of vitamin D3 supplementation on serum 25-hydroxyvitamin D (25(OH)D) levels. Notably, this analysis involved the systematic exclusion of studies with a loss to follow-up exceeding 15%. The included studies are labeled as follows: A/B. Close 2013a, C. Jastrzębska 2022, D. Jung, and E. Todd 2018. Close 2013a obtain two different dosage intervention groups.
